# Impact of Plasticizers on the Microbial Degradation of Polyhydroxybutyrate (PHB)

**DOI:** 10.3390/toxics14030194

**Published:** 2026-02-25

**Authors:** Yan Zhao, Yugo Matsumura, Peng-Cheng Zhao, Isha, Dubok Choi, Young-Cheol Chang

**Affiliations:** 1 Course of Chemical and Biological Engineering, Division of Sustainable and Environmental Engineering, Muroran Institute of Technology, Muroran 050-8585, Hokkaido, Japan; 25096008c@muroran-it.ac.jp (Y.Z.); 25041068v@muroran-it.ac.jp (Y.M.); 25096501m@muroran-it.ac.jp (I.); 2 Course of Chemical and Biological System, Department of Sciences and Informatics, Faculty of Science and Engineering, Muroran Institute of Technology, Muroran 050-8585, Hokkaido, Japan; 21026149@muroran-it.ac.jp; 3 Department of Science and Technology Convergence, Graduate School of Chosun University, Gwangju 61452, Republic of Korea; choidb@chosun.ac.kr

**Keywords:** PHB, additives, biodegradation, *Ralstonia* sp. C1

## Abstract

Polyhydroxybutyrate (PHB) is a biodegradable polyester considered a sustainable alternative to petroleum-based plastics. However, its biodegradation in the presence of plasticizers remains poorly defined. This study investigated the impact of phthalate ester- and glycol-based plasticizers on PHB degradation by *Ralstonia* sp. C1. Real Time -Polymerase Chain Reaction(RT-PCR) analysis showed that expression of the PHB depolymerase gene *phaZa1* remained unchanged in all additive-treated cultures, indicating no transcriptional interference. Liquid-medium degradation assays quantified by HPLC revealed rapid PHB utilization, with more than 50% degraded within 24 h and over 98% degraded within 48 h, with no significant differences relative to the control. Growth-inhibition assays further demonstrated that none of the plasticizers impaired bacterial viability, as OD_600_ profiles were comparable to untreated cultures. Soil degradation experiments confirmed that PHB films containing additives decomposed at rates similar to additive-free films, reaching approximately 80% degradation within 10 weeks. Overall, the tested plasticizers did not affect enzyme expression, microbial activity, or PHB biodegradation, highlighting the suitability of plasticized PHB materials for environmentally sustainable applications and supporting their scalable use as biodegradable alternatives to conventional plastics.

## 1. Introduction

Plastics have become a part of our daily lives, contributing to various aspects of daily life and industry since their development in the 19th century. These plastics are used worldwide owing to their low cost, light weight, durability, corrosion-resistance, and ease of processing [1,2]. Plastic production has increased annually, rising from 2 million tons per year in 1950 to 380 million tons per year by 2015, and currently stands at approximately 450 million tons annually [3,4]. While petroleum-based plastics are mass-produced and used extensively due to their usefulness, their highly stable physical properties mean that they remain largely intact in the environment when discarded and persist without significant degradation. Problems arise when marine species ingest these plastics, causing direct physical damage and potentially toxic effects [5]. While incineration and landfill disposal are common methods for plastic waste, the resulting emissions of greenhouse gases like carbon dioxide and harmful substances such as dioxins are also problematic. Furthermore, petroleum resources are finite, necessitating measures to consider the possibility of future depletion [6,7].

As a solution to these problems, polyhydroxyalkanoates (PHAs), a type of naturally biodegradable polyester produced by microorganisms, have gained attention in recent years [8]. PHA has environmentally friendly properties. It is converted into carbon dioxide and water by microorganisms in the environment under aerobic conditions, and into harmless substances such as methane under anaerobic conditions [8,9]. Furthermore, PHA possesses similar physicochemical, thermal, and mechanical properties to plastics such as polypropylene (PP) and low-density polyethylene (LDPE) [10]. Therefore, PHAs are expected to be a substitute for conventional plastics. To date, over 150 distinct PHA structures have been identified. Polyhydroxybutyrate (PHB) is the most common form of PHA. This is a homopolymer of 3-hydroxybutyrate (3-HB). PHB excels in barrier permeability and thermoplasticity (See Figure S1 in supplementary section). In addition, they possess high biocompatibility and are utilized in biomedical and packaging materials [10,11,12].

Although these PHA products are biodegradable, they can still adversely affect the environment until they degrade, which is similar to petroleum-based plastics. Therefore, there is an urgent need for their rapid biodegradation. Consequently, research investigating the degradation of these polymers in natural environments is important [13].

Plastic additives are commonly used in the manufacture of plastic products. They are added to impart necessary properties to the plastic polymer and facilitate the production process. For example, these include antioxidants to maintain the polymer matrix from oxidation, plasticizers to increase plastic flexibility, flame retardants to impart fire resistance, and processing aids (e.g., polymerization catalysts, solvents, lubricants) to enable or facilitate plastic manufacturing or processing [14].

Currently, chemical substances classified as plastic additives are approximately 10,000 types. Although the use of plastic additives makes it possible to create better products, they can cause serious damage to the environment and ultimately have adverse effects on human health if they accumulate in the natural environment. This is because some plastic additives cause endocrine disruption and reproductive toxic effects. For example, phthalate additives (PAEs) can cause infertility in men and early puberty in women, but these chemical substances are commonly used as plasticizers. Bisphenol A (BPA), used as a stabilizer, can disrupt the endocrine system, and polybrominated diphenyl ethers (PBDEs), used as flame retardants, can damage the respiratory system. Therefore, the use of plastic additives is often a cause for concern [15,16,17].

Additives are also used for the processing of biodegradable plastics. For example, plastics such as PLA and PHA have the drawbacks of slow crystallization rates and low crystallinity; therefore, nucleating agents are commonly added to promote crystallization. Other widely used additives include the antioxidants and plasticizers mentioned earlier, as well as antimicrobial agents that inhibit microbial activity [18]. These additives may have influenced the biodegradation of PHB. Extensive research has been conducted on the biodegradation of PHB; however, limited studies have discussed PHB biodegradation with plastic additives. These results indicate that the degradation rate varies depending on environmental conditions and polymer properties [19].

Some biodegradable plastic additives can enhance biodegradation by modifying the physical properties of PHB. However, non-biodegradable plastic additives may persist in the environment and inhibit the biodegradation process [19]. Therefore, gaining insights into the impact of plastic additives on PHB biodegradation is crucial for selecting appropriate additive types and usage levels in the future. In one study, Katarina Savva et al. have characterised the presence of plastic additives in single-use materials (collected from retail shops in Spain), which are made of the most common bio-based biodegradable materials, poly (lactic acid) (PLA) and poly(hydroxybutyrate) (PHB). An average of 123 plastic additives were found in PLA items and 121 in PHB items. Plasticisers are the most abundant additives; the phthalate group and glycol groups are the most commonly found [20]. Hence, it is crucial to understand the effects of phthalates and glycol plastic additives on PHB biodegradation.

This study aimed to investigate the effect of plastic additives such as phthalates and glycols on the PHB biodegradability of the previously isolated bacteria *Ralstonia* sp. C1 [21]. The bacterial strain *Ralstonia* sp. C1 used in this study was previously isolated from Mt. Kurodake in Daisetsuzan National Park, Hokkaido, and possesses PHB degradation capability [21]. Its PHB degradation capacity is the highest among all strains isolated to date in our laboratory. In soil degradation of PHB film at 30 °C, complete degradation within 20 days was confirmed upon addition of *Ralstonia* sp. C1. Furthermore, it was confirmed that this strain exhibits strong environmental adaptability, maintaining PHB degradation capability across a wide range of conditions: temperatures from 14 °C to 50 °C and pH values from 4.0 to 10.0.

Four phthalate additives were selected: bis(2-ethylhexyl) phthalate (DEHP), diallyl phthalate (DAP), dibutyl phthalate (DBP), and diethyl phthalate (DEP) and four glycol-based additives were selected: polyethylene glycol (PEG), diethylene glycol (DEG), di-propylene glycol (DPG), and butyl di-glycol acetate (BDGA) (for chemical structures see Figure S1 in supplementary section). Although these plastic additives have been found to be incorporated in PHB films to enhance their properties, there is limited knowledge about the effect of these additives on PHB biodegradability [20]. To the best of the authors’ knowledge, this is the first study to investigate the impact of these plastic additives on PHB biodegradation. This study plays a crucial role in the selection of plastic additives for sustainable bioplastic waste management.

## 2. Materials and Methods

### 2.1. Real-Time PCR Analysis of phaZa1 Gene Expression in Ralstonia sp. C1 in the Presence of Phthalate- and Glycol-Based Additives

*Ralstonia* sp. C1 was cultured in MS medium with polyhydroxybutyrate (PHB; Sigma-Aldrich, Tokyo, Japan; 0.5%, *w*/*v*) with separate individual additives, and a culture without additives was used as a control. The additives were bis(2-ethylhexyl) phthalate (200 µg/L), diallyl phthalate (200 µg/L), dibutyl phthalate (1000 µg/L), diethyl phthalate (500 µg/L), polyethylene glycol (2000 µg/L), diethylene glycol (2000 µg/L), dipropylene glycol (2000 µg/L), and butyl di-glycol acetate (2000 µg/L), respectively. Cultures were incubated at 30 °C and allowed to induce the expression of the *phaZa1* gene that encodes PHA depolymerase, after 24 h. The cells were collected through centrifugation at 5000 rpm for 10 min at 4 °C. The protocol used by the manufacturer of TRIzol reagent was followed, and a UV-visible spectrophotometer was used to estimate total RNA [22,23]. The total RNA was synthesized as complementary DNA (cDNA) at 42 °C with the PrimeScript II 1st strand cDNA synthesis kit and inactivated at 95 °C with the enzyme. Real-time PCR was done in a 20 µL reaction volume with SYBR Premix Ex Taq. The amplification conditions included a pre-treatment at 95 °C, 10 min and 30 cycles of 95 °C, 60 °C, and 72 °C, 30 s, 40 s, and 60 s, respectively. Primer specificity of the primers was verified by melting curve analysis (65–95 °C). The expression of *phaZa1* was normalized to that of the 16S rRNA gene as a housekeeping gene. Each experiment was performed in triplicate, and three independent biological replicates were done [24,25].

### 2.2. Test Strain and Preparation of PHB-Based Passage, Pre-Culture, and Primary Culture Media

In this study, *Ralstonia* sp. strain C1, a Gram-negative rod isolated from Mt. Kurodake in Daisetsuzan National Park, Hokkaido, Japan, was used to evaluate the biodegradation of PHB containing plasticizers. Members of the genus *Ralstonia* have been reported to possess the ability to synthesize and degrade PHB [17]. The strain was maintained by weekly passage on 0.1% (*w*/*v*) PHB agar medium. For pre-culture preparation, a single colony of *Ralstonia* sp. C1 was inoculated into 100 mL of Luria–Bertani (LB) liquid medium and incubated to obtain sufficient biomass. The primary culture medium consisted of 70 mL Minimal Salt (MS) medium supplemented with plastic additives at concentrations (Table 1) [18]. Water-insoluble additives (phthalates) were dissolved in an appropriate organic solvent (acetone < 1%) prior to addition. PHB powder (0.35 g) was added to obtain a final concentration of 0.5% (*w*/*v*), emulsified by sonication, and thoroughly mixed. The prepared medium was dispensed into sterile vials and used in subsequent biodegradation experiments.

The concentrations of plastic additives were selected on the basis of their reported occurrence levels in the study by Savva et al. (2023) [20].

### 2.3. PHB Degradation Experiment and Measurement of Residual PHB Under Additive Conditions

To ensure that PHB served as the sole carbon source, cells from an 18 h shaking culture were harvested and washed to remove residual nutrients. For this purpose, the culture broth was centrifuged at 10,000 rpm for 5 min at 4 °C. The supernatants were discarded, and the cell pellets were resuspended in sterile MS liquid medium, followed by a second centrifugation under the same conditions. After removal of the supernatant, sterile MS medium was added to each pellet to a final volume of 11 mL per tube. The suspensions were combined into a single 50 mL tube to obtain a homogeneous bacterial suspension.

For inoculation, 1 mL of the prepared cell suspension was added to 0.5% (*w*/*v*) PHB liquid medium containing plastic additives, resulting in a total culture volume of 20 mL and a final inoculum concentration of 5% (*v*/*v*). Control cultures received 1 mL sterile MS medium instead of the bacterial suspension. All cultures were incubated aerobically at 30 °C with shaking at 150 rpm for 96 h. Samples for residual PHB analysis were collected at 24 h intervals by transferring 1 mL of culture into microcentrifuge tubes containing 0.1 mL of phosphoric acid (Kanto Chemical Co., Inc., Tokyo, Japan) to terminate further degradation.

Residual PHB content was quantified following the method of Saito et al. [26] using a high-performance liquid chromatography (HPLC) system (Shimadzu Corporation, Kyoto, Japan). Samples were centrifuged, washed with ultrapure water, dried at 120 °C, and subjected to alkaline digestion with 5 N NaOH (FUJIFILM Wako Pure Chemical Corporation, Osaka, Japan) at 120 °C for 30 min. After cooling, the pH was adjusted to 3.0, the volume was brought to 10 mL, and the solution was filtered and diluted prior to HPLC analysis. The operating conditions are summarized in Table 2 [26].

### 2.4. Growth Inhibition Assay on Ralstonia sp. C1 Using the Highest Concentrations of Pthalate- and Glycol-Based Additives

A growth inhibition assay was conducted in Luria–Bertani (LB) medium to evaluate the effects of common plastic additives on the growth of *Ralstonia* sp. C1 under nutrient-rich conditions [27,28,29,30]. Stock solutions of each additive were prepared, dissolving the hydrophobic compounds in acetone and ensuring that the final solvent concentration in the cultures did not exceed 1% (*v*/*v*). The additives were tested at three concentrations, consistent with previously established PHB-related experiments, with specific working concentrations of bis(2-ethylhexyl) phthalate (200 µg/mL), diallyl phthalate (200 µg/mL), dibutyl phthalate (1000 µg/mL), diethyl phthalate (500 µg/mL), polyethylene glycol (2000 µg/mL), diethylene glycol (2000 µg/mL), dipropylene glycol (200 µg/mL), and butyl diglycol acetate (2000 µg/mL). Bacterial inocula were prepared by cultivating *Ralstonia* sp. C1 overnight in LB at 30 °C and 150 rpm, harvesting cells by centrifugation (10,000 rpm, 5 min), resuspending them in fresh medium, and adjusting the starting cell density to OD_600_ 0.08–0.11. For each condition, 20 mL cultures were assembled in sterile tubes containing LB medium, the appropriate additive concentration, and 100 µL of inoculum, with controls including LB-only, solvent control, and bacteria-only groups. All experiments were performed in triplicate. Cultures were incubated at 30 °C with shaking (150 rpm) for 24–48 h. Bacterial growth was monitored by measuring OD_600_ at 0, 2, 4, 8, 12, 24, and 48 h, using LB or LB + solvent (ace as blank as appropriate). Growth curves (OD_600_ vs. time) were plotted and reported as mean ± standard deviation. Additives producing OD_600_ values comparable to the untreated control were interpreted as displaying no measurable inhibitory effect on bacterial growth [31,32,33].

### 2.5. PHB Soil Decomposition Experiment

PHB films were prepared using the solvent casting method as previously described [33]. Briefly, PHB powder (0.6 g; Sigma-Aldrich, Tokyo, Japan) and plastic additives, polyethylene glycol and bis(2-ethylhexyl) phthalate, were dissolved in 30 mL of chloroform (FUJIFILM Wako Pure Chemical Corporation, Osaka, Japan) at concentrations corresponding to the maximum levels listed in Table 1. The solution was agitated and incubated at 60 °C for 2 h to ensure complete dissolution. The homogeneous mixture was poured into a 9 cm glass Petri dish and allowed to evaporate under ambient conditions in a fume hood. After solvent evaporation, the PHB film was recovered, cut into 1.5 × 1.5 cm^2^ pieces (0.02 g each), and sterilized with 70% ethanol (Kanto Chemical Co., Inc., Tokyo, Japan) followed by UV irradiation for 30 min. Soil samples were collected from the campus grounds of the Muroran Institute of Technology and sieved through a 14-mesh sieve to obtain a uniform particle size. The soil was air-dried and sterilized by heating at 121 °C for 3 h. Sterility was confirmed by incubating the treated soil in nutrient broth and potato dextrose broth at 30 °C for 7 days, during which no microbial growth was observed. A soil biodegradation experiment was conducted using sterilized soil inoculated with *Ralstonia* sp. C1. Approximately 40 g of soil was placed in a 50 mL polypropylene tube, and 10 mL of bacterial suspension prepared in Minimal Salt Medium was added and mixed thoroughly. A sterilized PHB film was then introduced, and the tube was sealed with a gas-permeable silicone stopper. The system was incubated at 30 °C, with soil moisture maintained at 45–55%. At 7-day intervals, PHB films were recovered, washed, dried, and weighed. All experiments were conducted in triplicate.

$$Degradation\;rate\left(\%\right)=\left[\frac{W_{1}-W_{2}}{W_{1}}\right]\times 100$$

W_1_: Initial weight of the PHB film (0.02 g), W_2_: Final weight of the buried PHB film remaining after the given degradation time.

## 3. Results

### 3.1. The phaZ1 Gene Expression Using Real-Time PCR

**Figure 1:**
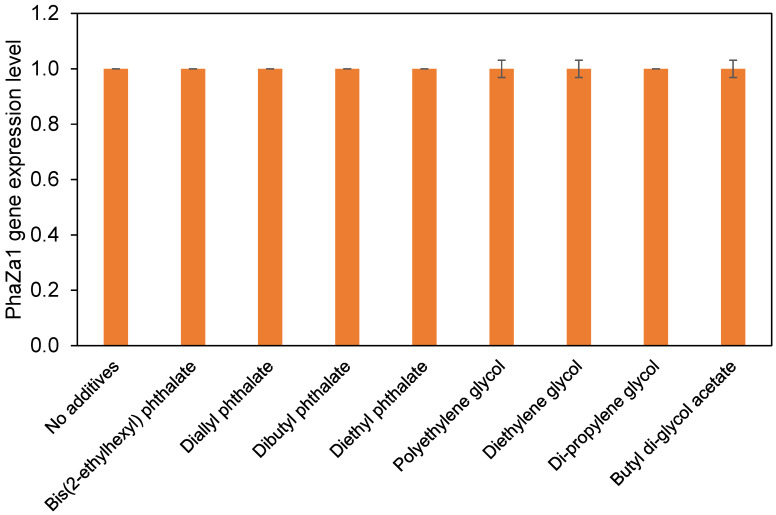
Relative expression of the *phaZa1* gene in *Ralstonia* sp. C1 grown in MS medium with 0.5% (*w*/*v*) PHB and different additives. The culture without additives was used as the control (set to 1.0). Gene expression was quantified by RT-PCR after 24 h at 30 °C and normalized to 16S rRNA. The values represent the mean of three biological replicates ± SD.

Figure 1 shows the relative expression of the *phaZa1* gene in *Ralstonia* sp. C1 during growth on PHB-containing MS medium using various individual additives. Overall, the levels of PhaZa1 expression in the control culture in the absence of additives were standardized to 1.0. The introduction of phthalate esters such as bis(2-ethylhexyl) phthalate, diallyl phthalate, dibutyl phthalate, and diethyl phthalate into the cultures did not result in any significant induction or suppression of PhaZa1 transcription relative to the control. On the same note, glycol additives like polyethylene glycol, diethylene glycol, di-propylene glycol, and butyl di-glycol acetate showed the same case; they yielded the same expression as that of the untreated culture. There were small differences between treatments, but this was within the range of errors and was not significant. The results suggest that, under the conditions and concentrations tested, none of the additives had a substantial effect on the expression of PhaZa1 after 24 h of growth.

### 3.2. Effect of Additives on PHB Degradation by Ralstonia sp. C1 in Liquid Medium

#### 3.2.1. The Impact of Phthalate Ester Additives on *Ralstonia* sp. C1 Strain

**Figure 2:**
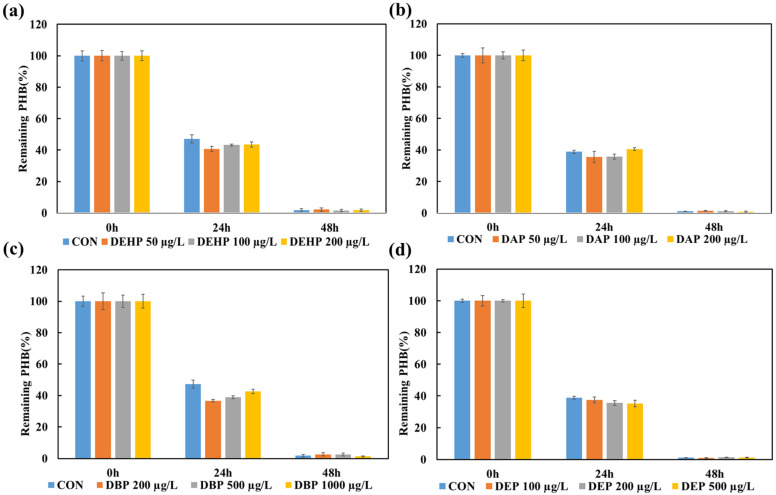
Residual mass of PHB samples as an indicator of biodegradation in the presence of phthalate-based additives, as compared to the control (CON) without additives. Time-dependent changes in PHB degradation under conditions with (**a**) bis(2-ethylhexyl) phthalate (DEHP), (**b**) diallyl phthalate (DAP), (**c**) dibutyl phthalate (DBP), and (**d**) diethyl phthalate (DEP). Values represent the mean of three biological replicates ± SD.

The degradation of PHB in the presence of different plastic additives was quantitatively compared to that of the control (no additive) at each time point (Figure 2). After 24 h, the control sample without plastic additives retained approximately 47% of its PHB. In comparison, DEHP-treated samples retained approximately 40% (50 μg/L), 43% (100 μg/L), and 44% (200 μg/L) of PHB. DAP exposure resulted in lower (non-significant) PHB retention compared with the control, with approximately 36% (50 μg/L), 37% (100 μg/L), and 40% (200 μg/L) PHB. DBP-treated samples showed PHB contents of approximately 36% (200 μg/L), 39% (500 μg/L), and 43% (1000 μg/L). In contrast, DEP-treated samples retained approximately 38% (100 μg/L), 36% (200 μg/L), and 35% (500 μg/L) of PHB.

After 48 h, the control retained approximately 2.0% PHB. DEHP-treated samples showed a PHB remaining of approximately 1.2–1.8%. The DAP-treated samples retained approximately 1.3–2.1%. DBP exposure resulted in slightly higher residual PHB at higher concentrations, approximately 2.5–3.0%, whereas DEP-treated samples retained approximately 0.8–1.5%. However, all the groups showed equal degradation after 72 h (See Supplementary Figure S2).

#### 3.2.2. The Effects of Glycol-Based Additives on *Ralstonia* sp. C1 Strain

The degradation of PHB in the presence of different plastic additives (DEG, PEG, DPG, and BDGA) was quantitatively assessed relative to that of the control (no additive) at each time point. At 0 h, the PHB content in the control and additive-treated samples was 100%. These results indicated no significant initial differences in PHB content across treatments.

After 24 h, the control retained approximately 47% of its PHB. The DEG-treated samples showed slightly lower retention, with 42% (200 µg/L), 41% (500 µg/L), and 40% (2000 µg/L). The PEG-treated samples retained 46%, 45%, and 44% PHB at 200, 500, and 2000 µg/L. In contrast, DPG exposure resulted in more pronounced degradation, with PHB retention of 32% (50 µg/L), 31% (100 µg/L), and 30% (200 µg/L). The BDGA-treated samples retained more PHB than the control, with values of 50% (200 µg/L), 52% (500 µg/L), and 55% (2000 µg/L).

At 48 h, the control contained approximately 2.5% PHB. The DEG-treated samples showed slightly lower retention, with 2.0%, 1.8%, and 1.5% PHB remaining at 200, 500, and 2000 µg/L. The PEG-treated samples retained 2.2, 2.0, and 1.8% PHB. The DPG-treated samples contained 2.0%, 1.8%, and 1.5% PHB. However, the BDGA-treated samples had slightly more PHB, with 3.0% (200 µg/L), 3.2% (500 µg/L), and 3.5% (2000 µg/L).

**Figure 3:**
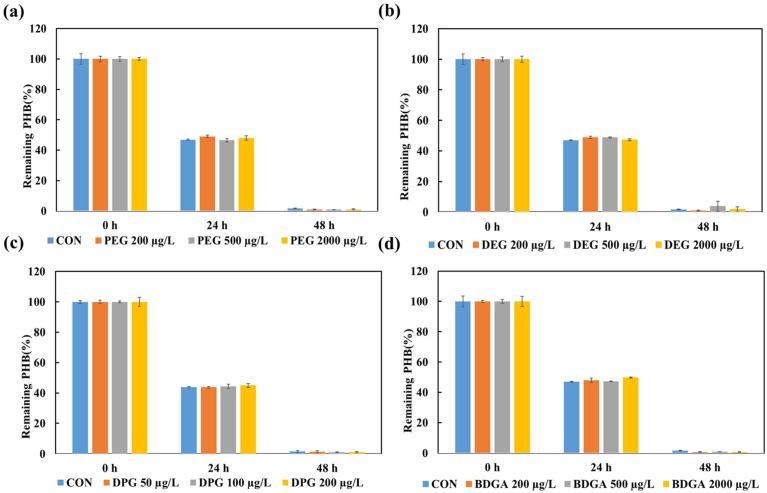
Residual mass of PHB samples as an indicator of biodegradation in the presence of glycol-based additives, as compared to the control (CON) without additives. Time-dependent changes in PHB degradation under (**a**) polyethylene glycol (PEG) conditions, (**b**) conditions with diethylene glycol (DEG), (**c**) conditions with dipropylene glycol (DPG), and (**d**) conditions with butyl di-glycol acetate (BDGA). Values represent the mean of three biological replicates ± SD.

By 72 h, the PHB remaining in the control had declined to approximately 0.6%. The DEG- and PEG-treated samples retained 0.5–0.3% PHB. The DPG-treated samples retained 0.4–0.3% of their initial weight. The BDGA-treated samples retained slightly more PHB, with values of 0.7%, 0.6%, and 0.8% at increasing concentrations (Figure 3).

### 3.3. Inhibition of Ralstonia sp. C1 Strain Growth by the Addition of Plasticizers

#### 3.3.1. Inhibition of *Ralstonia* sp. C1 Strain Using Phthalate Ester Additives (Evaluation by OD_600_)

**Figure 4:**
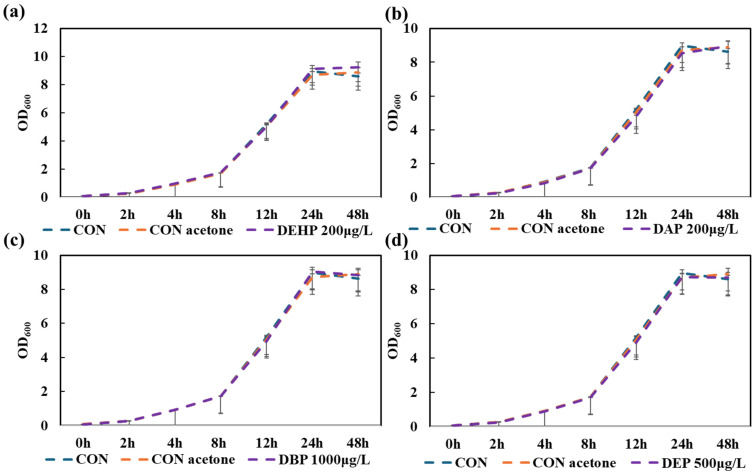
Time-dependent shifts in the OD_600_ of *Ralstonia* sp. C1 under conditions in which phthalate ester additives were added, along with LB media, compared to a control (CON) without additives (only LB medium) and a control growth medium with acetone (CON acetone) for 48 h. (**a**) Time-varying shifts in OD_600_ in the presence of bis(2-ethylhexyl) phthalate (DEHP). (**b**) OD_600_ over time in the absence of diallyl phthalate (DAP). (**c**) OD_600_ over time in the absence of dibutyl phthalate (DBP). (**d**) OD_600_ over time in the presence of diethyl phthalate (DEP). Values represent the mean of three biological replicates ± SD.

In the absence of PHB addition, a change in OD_600_ over time was observed in LB liquid medium, in which DEHP (200 µg/L), DAP (200 µg/L), DBP (1000 µg/L) and DEP (500 µg/L) additives (maximum concentration) were added to check the toxic effect on *Ralstonia* sp. C1. Growth control of acetone (<1%) was also added to nullify its effect. Figure 4 shows that for all four additives, OD_600_ increased over time, indicating a trend of growing bacterial numbers. Additionally, the increased patterns matched those of the growth curve of the control group (LB liquid medium only, no additives). Hence, there was no significant change in the growth of *Ralstonia* sp. over time compared to the control.

#### 3.3.2. Inhibition of *Ralstonia* sp. C1 Strain Using Glycol-Based Additives (Evaluation by OD_600_)

**Figure 5:**
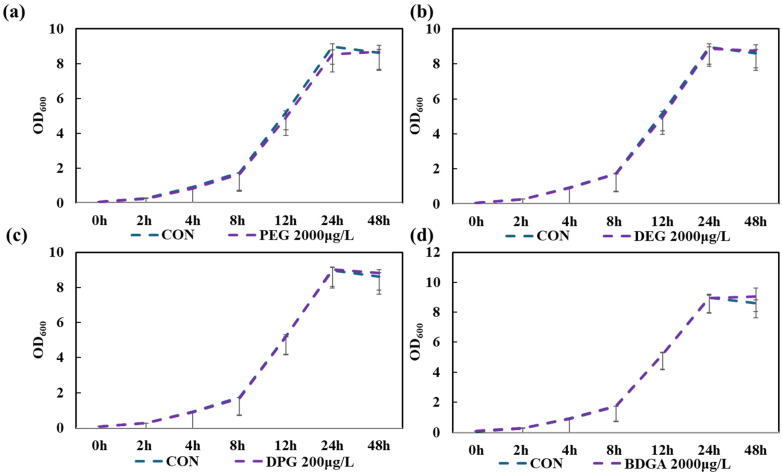
Time-dependent changes in OD_600_ of *Ralstonia* sp. C1 under conditions where glycol-based additives were the sole carbon source, compared to the control (CON) without additives. (**a**) Time-dependent changes in OD_600_ in the presence of polyethylene glycol (PEG). (**b**) OD_600_ over time in the presence of diethylene glycol (DEG). (**c**) OD_600_ over time in the presence of dipropylene glycol (DPG). (**d**) OD_600_ over time in the presence of butyl di-glycol acetate (BDGA). Values represent the mean of three biological replicates ± SD.

For glycol-based additives, OD_600_ was applied to detect bacterial strain change over time under the same experimental conditions, PEG (2000), DPG (2000), DEG (200), and BDGA (2000) (maximum concentration), as for phthalate ester-based additives. The results shown in Figure 5 indicate that for all additives, the bacterial count increased from 0 to 48 h. For more than 24 h, the difference between the additives group and the control group in OD_600_ over time remained within approximately 0.01, with no significant decreasing or increasing trend observed. Furthermore, these changes over time were found to be generally consistent with the control group (LB liquid medium only, no additives); that is, there was no significant difference in the growth of *Ralstonia* sp. C1.

### 3.4. Effect of Additives on PHB Degradation in Soil by Ralstonia sp. C1 Strain

**Figure 6:**
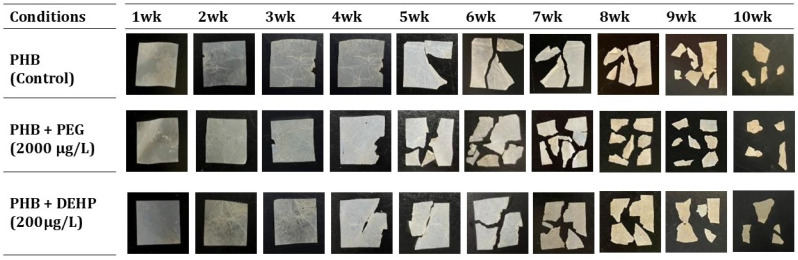
Physical changes (PHB film and PHB films with additives like PEG 2000 µg/L and DEHP 200 µg/L) due to biodegradation in soil via *Ralstonia* sp. C1 over time (week 1 to week 10).

To determine the effect of additives on PHB degradation in soil by *Ralstonia* sp. C1, one phthalate ester-based (DEHP) and one glycol-based (PEG) additive were chosen. PHB films with these additives were prepared, and experiments were done to study the time-dependent degradation of the *Ralstonia* sp. C1 strain in soil from the Muroran Institute of Technology campus. Photographs of the PHB film were taken from week 1 to week 10 in Figure 6, showing no major difference in the degradation rate between the control group (PHB film without additives) and the additive group.

**Figure 7:**
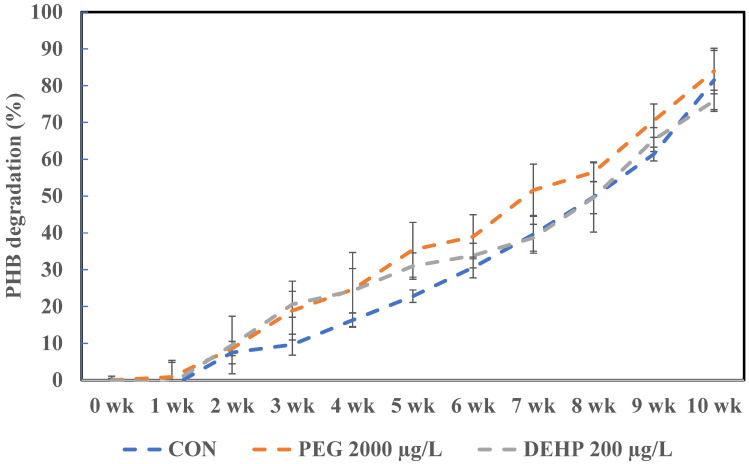
Graphical representation of the biodegradation percentage of PHB with additives compared to control (CON) without additives in sterile soil by adding *Ralstonia* sp. C1 is a sole microbe for degradation. Values represent the mean of three biological replicates ± SD.

Weighing the PHB films in the photographs and calculating the PHB film degradation rates shown in Figure 7 revealed that the PEG-added group seemed to degrade slightly faster than the control group, with a maximum difference of approximately 13% at week 7. However, by week 10, roughly 80% had degraded, similar to the control group. The DEHP-supplemented group decomposed slightly faster than the control group from week 2 to week 6, showing a maximum difference of approximately 11% in week 3. However, by week 10, the decomposition rate had also reached approximately 80%. Therefore, under conditions with additives, the *Ralstonia* sp. C1 strain showed a slight tendency to speed up the decomposition rate of PHB in the soil, but no significant difference was found compared to the control group.

## 4. Discussion

Plastic additives play a crucial role in improving the performance and applicability of bioplastics such as PHB. Although PHB is a biodegradable and environment friendly bioplastic, its inherent brittleness and narrow processing window limit its widespread use. Additives, such as plasticizers, are incorporated to enhance flexibility, toughness, and processability by reducing crystallinity and increasing polymer chain mobility. Therefore, identifying benign plasticizers that balance the mechanical performance with environmental compatibility (biodegradation) is essential for advancing sustainable bioplastic applications [34,35,36].

Plasticizers such as phthalate esters (DEHP) have been reported to possess toxicity, including carcinogenicity, reproductive toxicity, and respiratory effects, due to their long, branched alkyl side chains, which make them difficult to degrade in the natural environment. DBP and DEP have shorter alkyl chains and higher biodegradability than DEHP; however, they have been reported to exhibit reproductive toxicity [34,37,38,39,40]. Little research has been conducted on DAP biodegradability, and few toxicity assessments exist; it is known to exhibit hepatotoxicity and teratogenicity [41]. Therefore, there are two major concerns regarding the use of plasticizers, i.e., their effect on the biodegradability of bioplastic and their toxic effect on human physiology. This study focused on the former issue and used an efficient PHB-degrading strain (*Ralstonia* sp. C1) against phthalate esters and glycol-based PHB to check its biodegradation rate.

To determine the effects of additives on PHB-degrading *Ralstonia* sp. C1 bacteria, the expression level of the *phaZa1* gene was determined [21]. The unchanged PhaZa1 *expression* in *Ralstonia* sp. C1 across all tested additives indicates that phthalate esters and glycol-based plasticizers do not interfere with the transcriptional regulation of PHB depolymerase under the applied conditions. This suggested that PHB degradation by *Ralstonia* sp. C1 proceeds independently of these additives, and the presence of plasticizers neither induces stress responses nor suppresses the key biodegradation pathways. The lack of transcriptional modulation also supports the observation that the strain did not metabolize these compounds.

This study also evaluated PHB degradation by *Ralstonia* sp. C1 in both liquid medium and soil in the presence of phthalate ester and glycol-based additives. PHB degradation in liquid medium is shown in Figure 2, regarding phthalate ester additives, phthalic acid bis(2-ethylhexyl) (DEHP), phthalic acid diallyl (DAP), phthalic acid dibutyl (DBP), and phthalic acid diethyl (DEP), in addition to PHB. The data showed no significant difference in the degradation rate compared with the control group (PHB with no additives). The current study evaluated the toxicity of phthalates in liquid culture medium with a maximum concentration of 200 μg/L for DEHP, 200 μg/L for diallyl phthalate (DAP), 1000 μg/L for dibutyl phthalate (DBP), and 500 μg/L for diethyl phthalate (DEP), which did not significantly affect PHB degradation by *Ralstonia* sp. C1. The degradation of liquid medium by glycol-based additives is shown in Figure 3, and the results are similar to those of phthalate ester additives; the degradation rate of PHB by *Ralstonia* sp. C1, concentrations were devised from Katarina Savva et al. (2023) [20].

Among the four glycol-based additives used in this study, PEG is commonly used as a plasticizer in plastics and is considered to have low toxicity [42,43,44]. In contrast, DEG is primarily used as a processing aid and exhibits ecotoxicity [45,46]. Regarding DPG and BDGA, research on these two additives is limited. DPG itself does not function as a plasticizer; rather, its derivative dipropylene glycol dibenzoate (DPGDB) is commonly used as a plasticizer [47]. BDGA is also a glycol-based additive and is expected to serve as a plasticizer or processing aid [20]. Studies evaluating DPG and BDGA as additives are limited, and no conclusive evidence of clear toxicity has been reported.

Based on the results of PHB degradation in liquid medium, the concentrations used in this study—polyethylene glycol (PEG) 2000 μg/L, diethylene glycol (DEG) 2000 μg/L, dipropylene glycol (DPG) 200 μg/L, and butyl di-glycol acetate (BDGA) 2000 μg/L did not affect the PHB degradation of *Ralstonia* sp. C1 strain. Furthermore, all eight additives evaluated in this study were considered to be within a concentration range that is realistically achievable during the actual manufacturing of plastic products [20], corresponding to the maximum concentration examined in this study.

Several studies have investigated the biodegradability of PHB with additives. Read et al. added triethyl citrate (TEC) as a plasticizer to a PHBV film. They examined its biodegradability in marine environments and mesocosms and found that the biodegradation patterns were highly similar, regardless of the additive presence. They also evaluated a commonly used natural rigid filler (WF) to improve the mechanical properties of plastics. The results showed no change in biodegradability due to WF addition [48]. Other studies on WF have been conducted; for instance, Chan et al. reported that increasing the wood content in PHBV-WF composites enhanced their biodegradability in soil [49]. Pavel Brdlík et al. investigated the effects of the naturally derived plasticizer acetyl tributyl citrate (ATBC), a heterogeneous nucleating agent, calcium carbonate (CaCO_3_), and spray-dried lignin-coated cellulose nanocrystals (L-CNC) added to PHBV on biodegradability. Among the PHBV biocomposites subjected to composting, the ternary PHBV composite film containing 10 wt.% ATBC and CaCO_3_ exhibited the highest biodegradability. These studies suggest that the addition of additives likely alters the physical properties of PHBV films, making them more accessible to microbial degradation enzymes, which in turn leads to changes in biodegradability [50].

With regard to the toxicity of additives on bacterial growth in this study, the growth inhibition assay showed that none of the phthalate- or glycol-based additives suppressed the growth of *Ralstonia* sp. C1 at the highest tested concentrations. OD_600_ values increased consistently across all treatments, closely matching the LB-only control, indicating that the additives neither interfered with cellular metabolism nor imposed measurable stress. The minimal differences between the treated and untreated cultures also suggested that the additives were not utilized as alternative carbon sources. Overall, these results confirmed that the plasticizers examined are non-inhibitory to *Ralstonia* sp. C1 under nutrient-rich conditions, supporting the conclusion that PHB degradation performance is unaffected by additive-related toxicity.

As summarized in Table 3, for phthalate ester additives, *Pseudomonas* and *Ralstonia* strains can utilize DEHP, DBP, and DEP, and no toxic effect on the strains has been reported. For glycol-based additives, strains that can utilize PEG and DEG as carbon sources belong to the genera *Acinetobacter* and *Pseudomonas*. *Pseudomonas* is a representative PHB-degrading bacterium, and Acinetobacter has been reported as a PHB-degrading bacterium [51,52].

The other three additives (DAP, DPG, and BDGA) among the five listed in the table have been studied only in a limited manner, primarily in terms of their biodegradability, which remains unclear. Moreover, due to their similar properties within their respective groups, they are considered potentially usable as carbon sources. However, this study is limited to checking the toxicity of the additive using a growth inhibition assay.

Furthermore, this study evaluated the effects of additives not only on PHB degradation in liquid medium but also on degradation in soil environments. Table 4 summarizes several examples from previous studies on PHB film degradation in soil. The results showed that all groups, regardless of the presence of additives, exhibited approximately an 80% degradation rate over 10 weeks. This indicates that the additives do not affect the degradation of PHB by *Ralstonia* sp. C1 in soil. Furthermore, comparing the degradation results from this study with previous results on the soil degradation of PHB films, the degradation rate was slightly slower. This is likely due to the use of sterilized soil in this experiment. Chang et al. indicated that decomposition proceeds significantly faster in non-sterilized soil than in sterilized soil. This is suggested as being due to the fact that the soil containing only the *Ralstonia* sp. C1 strain has a reduced ability to form biofilms compared with soil containing multiple bacterial species [21].

Based on the results of this study, it can be concluded that many of the plastic additives examined did not affect PHB-degrading bacteria, and their impact on PHB degradability in the environment was limited. These findings are considered useful as foundational data for developing PHB products that reduce the environmental impact. Therefore, a detailed investigation of biodegradation behavior under conditions requiring high-concentration addition is necessary. This is expected to yield design guidelines for PHB products aimed at further reducing their environmental impact.

## 5. Conclusions

This study demonstrated that commonly used phthalate- and glycol-based plasticizers do not interfere with the biodegradation of polyhydroxybutyrate (PHB) by *Ralstonia* sp. C1. Across all tested concentrations, PHB degradation proceeded rapidly in liquid medium, with more than 50% degraded within 24 h and over 98% within 48 h, showing no significant differences from the control. RT-PCR analysis confirmed that the expression of the PHB depolymerase gene *phaZa1* remained unchanged in the presence of all additives, indicating that depolymerase regulation is unaffected by plasticizers. Growth-inhibition assays further showed that none of the plasticizers suppressed bacterial viability. Soil assays demonstrated comparable degradation rates between plasticized and additive-free PHB films. Collectively, these results show that the tested plasticizers do not affect enzyme expression, microbial growth, or PHB degradation, supporting the environmental suitability of plasticized PHB materials. Future work should evaluate degradation under mixed-microbial communities and assess additional additive categories.

## Figures and Tables

**Table 1:** Varied concentrations of different additives used in PHB degradation via *Ralstonia* sp. C1.

	Name of Additives	Concentration μg/L		
1.	Bis(2-ethylhexyl) phthalate (DEHP)	50	100	200
2.	Diallyl phthalate (DAP)	50	100	200
3.	Dibutyl phthalate (DBP)	200	500	1000
4.	Diethyl phthalate (DEP)	100	200	500
5.	Polyethylene glycol (PEG)	200	500	2000
6.	Diethylene glycol (DEG)	200	500	2000
7.	Di-propylene glycol (DPG)	50	100	200
8.	Butyl di-glycol acetate (BDGA)	200	500	2000

**Table 2:** The optimum conditions for HPLC for PHB content quantification.

Mobile Phase	860 μl/L perchloric acid (Kanto Chemical Co., Inc., Tokyo, Japan)
Flow Rate (mL/min)	1.5
Analysis Time	15 min
Column	SHIMADZU, SCR-102H, Kyoto, Japan
Column Temperature (°C)	45
UV-VIS Detector	SHIMADZU, SPD-20A, Kyoto, Japan
Detection Wavelength (nm)	210
Sample Injection Volume (μL)	5

**Table 3:** Additive biodegradability chart with different bacteria found in the literature.

Strain	Additive	Reference
*Pseudomonas fluorescens* FS1	DEHP, DEP	[53]
*Pseudomonas* sp. PS1	DBP	[54]
*Ralstonia pickettii*	DEP	[55]
*Acinetobacter* SC 25	PEG200, PEG400, DEG	[56]
*Pseudomonas* KW 8	PEG400	[56]

**Table 4:** PHB film soil degradation with respective microbial communities.

PHB Form	Microorganisms	Biodegradation Rate	Reference
Film	Soil microbial community	Within three weeks, all of the PHB nanofibers were broken down, and in six weeks, 62% of the PHB film was broken down.	[33]
Film	Soil microbial community	82% of PHB was broken down over the course of the 80-day degradation period.	[57]
Film	*Rhisobiaceae*, *Pseudomonaceae*, *Alcaligenaceae*, *Shingobacteriaceae*, *Bionectriaceae*, *Ophiocordycipitaceae*	95.7% of the PHB film deteriorated over the course of the 90-day degradation period.	[58]

## Data Availability

The original contributions presented in this study are included in the article. Further inquiries can be directed to the corresponding author.

## Supplementary Materials

The following supporting information can be downloaded at https://www.mdpi.com/article/10.3390/toxics14030194/s1, Figure S1. Chemical Structures of PHB, Phthalate Based Additive and Glycol Based Additives. Figure S2. HPLC calibration curve and chromatogram of 2-BE.
