# Supplementary material for: Impact of Plasticizers on the Microbial Degradation of Polyhydroxybutyrate (PHB)
Source: Toxics. 2026 Feb 25;14(3):194. doi: 10.3390/toxics14030194 (PMC13030448; doi:10.3390/toxics14030194)
Supplement: Supplementary file 1 [file toxics-14-00194-s001.zip › toxics-4132435-supplementary.pdf]

Supplementary Section

Impact of Plasticizers on the Microbial Degradation of Polyhydroxybutyrate (PHB)

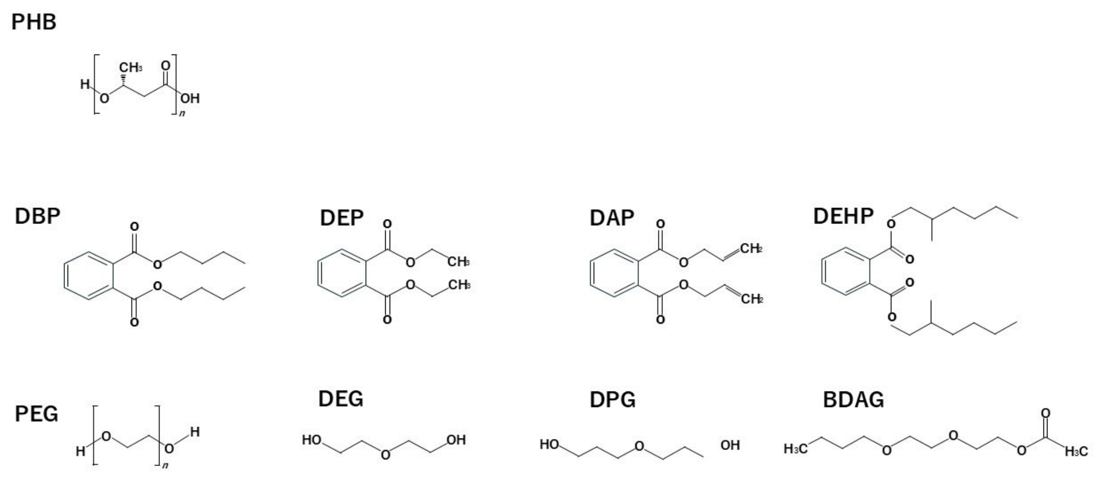

Figure S1. Chemical Structures of PHB, Phthalate Based Additive and Glycol Based Additives.

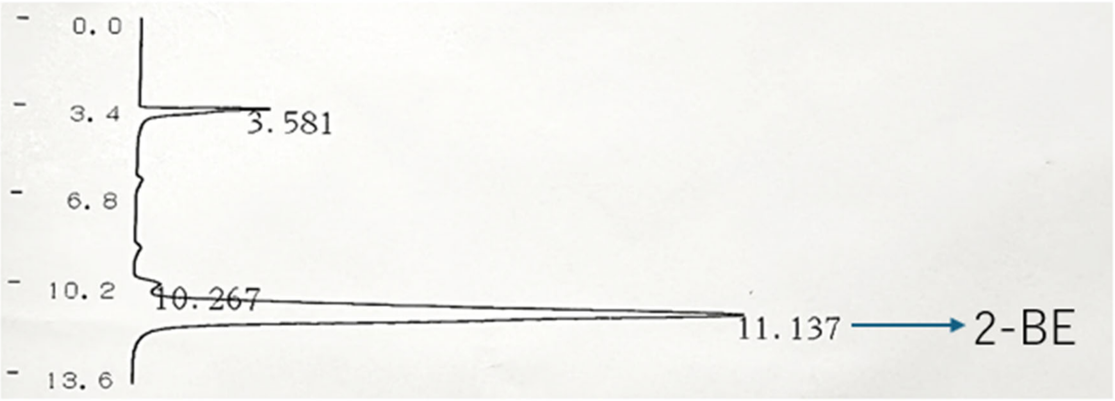

Figure S2. HPLC calibration curve and chromatogram of 2-BE.
